# Lifelong learning of Chinese rural physicians: preliminary psychometrics and influencing factors

**DOI:** 10.1186/s12909-015-0460-9

**Published:** 2015-10-30

**Authors:** Honghe Li, Ziwei Wang, Nan Jiang, Yang Liu, Deliang Wen

**Affiliations:** 1 School of Public Health, China Medical University, Shenyang, Liaoning Province China; 2 Research Center for Medical Education, China Medical University, Shenyang, Liaoning Province China; 3 School of Public Health, Dalian Medical University, Dalian, Liaoning Province China

**Keywords:** Lifelong learning, Rural physicians, Professionalism, Continuing medical education

## Abstract

**Background:**

There are more than 4.9 million rural health workers undertaking the health care need of rural population of over 629 million in China. The lifelong learning of physicians is vital in maintaining up-to-date and qualified health care, but rural physicians in many developing countries lack adequate medical professional developments. There has also been no empirical research focused on the lifelong learning of rural physician populations. The purpose of this study was to investigate the primary levels of lifelong learning of the rural physicians and to analyze group differences.

**Methods:**

We conducted a cross-sectional study on 1197 rural physicians using the Jefferson Scale of Physician Lifelong Learning (JSPLL). Cronbach’s α coefficient, exploratory factor analysis, independent sample *t*-test, and one-way ANOVA followed by Student-Newman-Keuls test were performed to analyze the data.

**Results:**

For Chinese rural physicians, the JSPLL was reliable (Cronbach’s α coefficient = 0.872) and valid, with exploratory factor analysis fitting a 3-factor model and accounting for a total of 60.46 % of the variance. The mean lifelong learning score was 45.56. Rural physicians generally performed worse in the technical skills in seeking information domain. Rural physicians with 21-30 working years have a lower score of lifelong learning (*P* < 0.05) than other phases of working years. Career satisfaction and professional titles had a significantly positive influence on physicians’ orientation towards lifelong learning (*P* < 0.05). The overall lifelong learning scores of physicians who received more training after completion of medical school were higher than those with less additional post-medical school training (*P* <0.05).

**Conclusions:**

The JSPLL is effective for the Chinese rural physician population. In order to cope with impacting factors on rural physicians’ lifelong learning, the results of the study reinforced the importance of continuing medical education and career satisfaction for lifelong learning and the need for medical schools and hospitals to provide reasonable strategies and necessary support for rural physicians with different amounts of working years. Providing rural physicians more educational opportunities and helping them access educational resources may be an effective strategy for improving their orientation to lifelong learning.

**Electronic supplementary material:**

The online version of this article (doi:10.1186/s12909-015-0460-9) contains supplementary material, which is available to authorized users.

## Background

China has a large rural population, surpassing 629 million in the year 2013 and accounting for 46.32 % of China’s total population [1]. Recently, rapid improvements in the level of living of rural residents, the change of disease spectrum from infectious disease to chronic disease, and the rapidly growing proportion of the senior population are all affecting the capacity of rural physicians to meet their patients’ higher expectations of health care [2, 3]. Historically, Chinese rural doctors have made great contributions to the primary health care of rural areas, covering villages, towns and counties throughout rural China [4]. However, the majority of rural physicians only received a 3 year medical education [5, 6] and lacked access to updates in medical knowledge and skills, so it is difficult for them to meet the ever increasing health care needs of rural residents [2, 3, 5, 7]. This resulted in urban tertiary referral hospitals^1^ being the first point of contact for a large number of rural patients, and this congestion led to poor access to medical care services and tense physician-patient relationships for both urban and rural patients [8–10]. Therefore, rural physicians must make a sustained effort to keep up-to-date with medical and continuing scientific developments in their relevant areas of expertise to improve the quality of their health services. Many other developing countries and regions, such as Saudi Arabia [11], sub-Saharan Africa [12–15], and Brazil [16], also faced similar situations and challenges in their rural health care sector. Rural physicians in many developing countries usually lack access to continued medical education to develop their medical competence and to keep up-to-date with current medical developments [2, 5, 13–17]. The lifelong learning of physicians plays a vital role in the above by maintaining qualified health care and providing a continuous improvement in medical practice and has obtained increasing attention from both health care educators and providers in recent years [18–21].

Lifelong learning, defined by Hojat et al in 2003 [21], is extremely valued in the medical practice. It has been described as an indicator of competence and professionalism and as a necessary requirement of being a qualified doctor [20, 22]. The need to cultivate lifelong learning in physicians is recognized by several national and international medical organizations [23–25]. The Chinese National Educational Plan for Rural Physicians (2011-2020) identified the development of lifelong learning and continuing medical education for rural physicians as one of its most important strategies to enhance the quality of primary health care in rural areas [26]. Thus, it is essential to first explore the current levels of lifelong learning in China’s rural physicians in order to better strategize continuing medical education programs to cultivate lifelong learning skills and to continuously improve the quality of medical treatment [5, 20].

Assessing the orientation toward lifelong learning in the rural physician population can give insight to their learning beliefs and motivations, current lifelong learning conditions, and other relevant factors. The Jefferson Scale of Physician Lifelong Learning (JSPLL) was found to be the most suitable in measuring orientation toward lifelong learning in physicians [21, 27, 28]. Previous studies using the JSPLL have provided good psychometric properties in different medical populations, including physicians affiliated with the Jefferson Health System, medical students, and nurses [21, 27–31]. In China, the JSPLL has been administrated to physicians and nurses in urban tertiary referral hospitals [32–34], but the validity and reliability of the JSPLL on Chinese health care providers was not fully assessed prior to our study. Thus far, there has been no empirical research focusing on measuring the lifelong learning of the rural physician population and its influencing factors. Therefore, our aims were to assess the effectiveness of the JSPLL on Chinese medical practitioners and to use it as an instrument to measure the lifelong learning of Chinese rural physicians as a preliminary study for rural physicians in general.

Therefore, in this study, we examined the reliability and validity of the Chinese version of the JSPLL, measured rural physicians’ orientation toward lifelong learning, identified whether continuing medical education can impact physicians’ orientation to lifelong learning, and explored some other possible influencing factors on their orientation toward lifelong learning.

This allowed us to better understand the current condition of Chinese rural physicians’ lifelong learning and in turn provide some correlates and influencing factors to health policy makers, health care administrators, and medical educators to improve rural physicians’ lifelong learning. In the long run, promoting rural physicians’ lifelong learning will enhance their quality of health care in rural areas, relieve the congestion of urban health care centers, and improve physician-patient relationships [20, 22].

## Methods

### Study sample

The cross-sectional study was conducted from April to July of 2013. The Chinese version of the JSPLL was distributed to 1,500 rural physicians from five different regions of Liaoning Province in China, 300 per rural region. In order to provide a uniform representation of all the regions, we have selected a city from each of north, east, south, west, and central areas of the province, respectively. Randomized cluster sampling methods were used to select physicians. Their levels of education ranged from junior college vocational medical training to university medical education (including post-graduate studies). The primary target of the three-year college programs was to produce medical personnel for rural residents. An overview of China’s medical education system and its four main streams is presented in Additional file 1: Figure S1.

Gender, age, professional title, highest level of education, and years of working experience from the study sample were proportionally compared to the 2013 China Health Statistics Yearbook to determine representation of China’s overall rural physician population.

### Questionnaire

The JSPLL, developed by Mohammadreza Hojat in 2003 [21], specifically measures physicians’ orientations toward lifelong learning. Before administration, in order to ensure accuracy of the translation, the JSPLL was translated to Chinese and back-translated to English by 2 bilingual researchers from Dalian Medical University and China Medical University. The translated Chinese version of the JSPLL contained 14 items, with each being answered on a 4-point Likert-type scale; each item was positively worded and directly scored from 1 (strongly disagree) to 4 (strongly agree) [28]. The sum of all items gave the total score. Total scores could range from 14 to 56, with higher scores indicating greater orientation toward lifelong learning.

Physicians could consult the surveyors, who were fully trained, with regard to any uncertainties about the questionnaire at any given time. The questionnaire had to be completed independently by the physician within 15 min. Participation was voluntary and participants had the ability to be selective about the questions they answered.

A socio-demographic questionnaire was also used. The questionnaire was confidential and was used to obtain information about gender, age, career satisfaction, educational levels, professional titles, years of employment, and professional training experience (Yes: participated in more than 15 days a year of continuing medical education; No: participated in less than 15 days a year of continuing medical education). Continuing medical education programs in China include training classes, senior courses, lectures, academic conferences and long-distance education programs held by the academic community and institutions which were approved by the National Continuing Medical Education Committee [35].

### Statistical analysis

Internal consistency of the JSPLL questionnaire for the entire assessment model was estimated by Cronbach’s α coefficient. Values equal to or greater than 0.70 were considered acceptable. To assess whether factor analysis could be conducted in this study, the Kaiser–Meyer-Olkin (KMO) analysis and Bartlett’s test of sphericity were performed. Exploratory factor analysis (principal component factor extraction and varimax rotation) was conducted to explore the factor structure of the data. Eigen values, relative magnitude and direction of factor loadings, were examined to explain variance and communality. Furthermore, lifelong learning scores from the study were compared to professional training experience and other possible factors with *t*-test and one-way ANOVA. Then, Student–Newman–Keuls test was used to make multiple comparisons between the different groups of years of employment.

Missing data were replaced with the median or mean values of the respective item. However, questionnaires missing demographic information and more than 20 % of the items were excluded from subsequent analysis. Domains were not scored when 20 % or more of the items were missing. The final analysis database was compiled after analytical treatment was performed to detect any logical errors and abnormal values. SPSS® version 17.0 (SPSS Inc., Chicago, IL, USA) for Windows® was used to analyze the data. A *P*-value of < 0.05 was considered to be statistically significant.

### Ethics statement

This study was approved by the Bioethics Advisory Commission of Dalian Medical University, Dalian, China. Written informed consents were obtained from the participants prior to having them take part in the study. In line with the terms of consent to which participants agreed, personal details were kept confidential for the study purpose only and not made publicly available. The assessment was self-administered, participation was voluntary, and participants were not compensated. All data in this study maintained anonymity, and numerical codes were assigned to participants.

## Results

### Socio-demographic characteristics of the population

1,197 of the 1,500 physicians (79.8 %) effectively completed and returned the questionnaire. Table 1 summarizes the social-demographics characteristics of the respondents.

**Table 1:** Demographic information on the survey sample of rural physicians

Demographic	Category	N (%)
Gender	Male	583 (48.7 %)
	Female	614 (51.3 %)
Age	21-30	52 (4.3 %)
	31-40	637 (53.2 %)
	41-50	321 (26.8 %)
	51-60	181 (15.1 %)
Professional title	Assistant physician	773 (64.6 %)
	Attending physician	379 (31.7 %)
	Associate chief physician	10 (0.8 %)
	Chief physician	1 (0.1 %)
Highest level of education	Junior college degree and below	1151 (96.2 %)
	Undergraduate degree and above	42 (3.5 %)
Years of work experience	5 years or less	111 (9.4 %)
	6-10 years	171 (14.5)
	11-15 years	306 (25.9 %)
	16-20 years	268 (22.7 %)
	21-25 years	150 (12.7 %)
	26-30 years	32 (2.7 %)
	More than 30 years	145 (12.3 %)
Career satisfaction	Very satisfied	187 (15.6 %)
	Relatively satisfied	547 (45.5 %)
	Neutral	347 (29 %)
	Relatively unsatisfied	92 (7.7 %)
	Very unsatisfied	18 (1.5 %)
Professional training experience	Yes	756 (63.2 %)
	No	391 (32.7 %)

The constituent ratios of gender, age, professional title, highest level of education, years of experience were similar to the China Health Statistics Yearbook [1] reports on social-demographic characteristics of rural physicians in 2003. Thus, the study sample could generally represent the overall rural physician population in China. More than half of the rural physicians (61.1 %) were satisfied with their career. Most of the rural physicians (63.2 %) participated in continuing medical education for more than 15 days a year.

### Reliability

The internal consistency of the JSPLL had an overall Cronbach’s α coefficient of 0.872, an acceptable range for educational and psychological testing as determined by professional testing organizations. In addition, each component's internal consistency reliability was assessed. Factor 1 (learning beliefs and motivation) showed an internal consistency reliability of 0.837, factor 2 (attention to learning opportunities) showed an internal consistency reliability of 0.754, and factor 3 (technical skills in seeking information) showed an internal consistency reliability of 0.663. Factor 1 and factor 2 show a high level of internal consistency reliability, suggesting that each component's items are measuring the main idea of the components.

### Construct validity

The Kaiser–Meyer-Olkin (KMO) analysis yielded an index of 0.93. The result for Bartlett’s test of sphericity was 5156.482, which is highly significant (*P* < 0.05). This information allowed us to proceed with exploratory factor analysis to identify the factor model. Summary results of factor analysis of the data for the 14 items are shown in Table 2.

**Table 2:** Exploratory factor analysis for the Chinese version of the Jefferson scale of physician lifelong learning

Item^a^	Rotated factor coefficients		
	Factor 1	Factor 2	Factor 3
1. Rapid changes in medical science require constant updating of knowledge and development of new professional skills.	**.77**	.23	.15
2. I believe that I would fall behind if I stopped learning about new developments in my profession.	**.73**	.12	.33
3. One of the important goals of medical school is to develop students’ lifelong learning skills.	**.70**	.08	.27
4. Searching for the answer to a question is, in and by itself, rewarding.	**.66**	.35	-.07
5. Lifelong learning is a professional responsibility of all physicians.	**.66**	.33	-.00
6. I recognize my need to constantly acquire new professional knowledge.	**.64**	.45	.14
7. I routinely attend annual meetings of professional medical organizations	.14	**.74**	.24
8. I routinely attend continuing medical education programs to improve patient care.	.29	**.70**	.21
9. I take every opportunity to gain new knowledge/skills that are important to my profession.	.32	**.69**	.29
10. I always make time for self-directed learning, even when I have a busy practice schedule and other professional and family obligations.	.29	**.59**	.40
11. I enjoy reading articles in which issues of my professional interest are discussed.	.28	**.57**	.29
12. I read professional journals at least once every week.	.08	.23	**.82**
13. I routinely search computer databases to find out about new developments in my specialty.	.15	.24	**.75**
14. My preferred approach in finding an answer to a question is to search the appropriate computer databases.	.15	.28	**.63**
% Variance	23.63	20.54	16.29

^a^Items are listed in order of magnitude of the factor coefficient within each factor. Values greater than 0.50 are in **bold**

As shown in Table 2, 3 factors emerged and accounted for a total of 60.46 % of the variance. Factor 1, a grand factor of “learning beliefs and motivation”, was based on 6 items with factor coefficients greater than 0.50 and accounted for 23.63 % of the variance. Factor 2, “attention to learning opportunities,” accounted for 20.54 % of the variance, with 5 items having coefficient factors greater than 0.50. Factors 3, “technical skills in seeking information,” consisted of 3 items and accounted for 16.29 % of the variance.

### Rural physicians’ orientation to lifelong learning

The mean, standard deviation, quartile points, and the range of actual scores of the total JSPLL and the mean scores of 3 domains are presented in Table 3.

**Table 3:** Descriptive statistics for the Chinese version of the JSPLL for rural physicians

Statistics	Value
Mean score of total scale	45.56
Standard deviation	6.17
25^th^ percentile	42
50^th^ percentile (median)	45
75^th^ percentile	50
Range	19-56
Mean score of factor 1	20.07
Mean score of factor 2	16.14
Mean score of factor 3	9.35

The item mean score is 3.35 for factor 1 “learning beliefs and motivation”, 3.23 for factor 2 “attention to learning opportunities”, and 3.12 for factor 3 “technical skills in seeking information”. Items in the "learning beliefs and motivation" domain has the highest mean score.

### Lifelong learning according to amount of continuing medical education

Rural physicians who took part in continuing medical education for more than 15 days a year obtained significantly higher scores than those who did not (*P* <0.01). The mean score of each domain in relation to the continuing medical education experience also showed significant differences (*P* < 0.05). In factor 1, only the scores of item 2 had significant differences (*P* < 0.01). In factor 2, scores of items 7, 8, 9, and 11 showed significant differences (*P* < 0.01). All of factor 3’s item scores had significant differences (*P* < 0.05). The scores of physicians with different amounts of continuing medical education are shown in Table 4.

**Table 4:** Scores of rural physicians with different amounts of continuing medical education

JSPLL domains	Continuing medical education experience (*mean* ± *SD*)		*t*	*P*-value
	More than 15 days	Less than 15 days		
Factor 1: learning beliefs and motivation	20.20 ± 2.95	19.74 ± 3.13	2.400	0.017
Factor 2: attention to learning opportunities	16.41 ± 2.32	15.50 ± 2.44	6.044	0.000
Factor 3: technical skills in seeking information	9.54 ± 1.54	8.95 ± 1.59	5.893	0.000
Total score	46.15 ± 6.06	44.19 ± 6.05	5.049	0.000

### Lifelong learning according to years of work experience

Lifelong learning of Chinese rural physicians was compared via one-way ANOVA according to different years of work experience (Table 5).

**Table 5:** Scores of rural physicians with different years of work experience

JSPLL domains	Years of work experience (*mean* ± *SD*)							*F*	*P-*value
	0-5	6-10	11-15	16-20	21-25	26-30	>30		
Factor 1	20.59 ± 2.35	20.15 ± 3.07	20.36 ± 2.74	19.89 ± 3.27	19.32 ± 3.56	19.31 ± 2.57	20.20 ± 2.87	3.131	.005
Factor 2	16.40 ± 2.13	16.11 ± 2.38	16.22 ± 2.30	16.00 ± 2.39	15.68 ± 2.65	15.50 ± 2.21	16.68 ± 2.51	2.995	.007
Factor 3	9.59 ± 1.67	9.43 ± 1.60	9.39 ± 1.58	9.33 ± 1.56	8.98 ± 1.70	9.06 ± 1.29	9.41 ± 1.74	2.014	.061
Total score	46.58 ± 5.35	45.69 ± 6.22	45.98 ± 5.83	45.23 ± 6.36	43.99 ± 6.88	43.87 ± 5.28	46.29 ± 6.28	3.270	.003

**Fig. 1:**
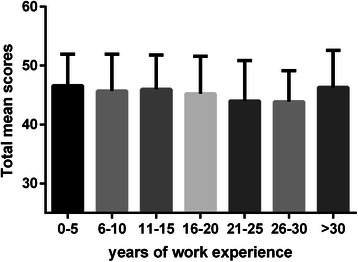
Total mean scores of lifelong learning relative to working years. Each bar indicates the total mean scores of rural physicians’ orientation toward lifelong learning relative to the different years of their work experience. The Y error bar was displayed on top of each bar. The Rural physicians with 0-30 working years showed significant impairment in orientation toward lifelong learning

**Fig. 2:**
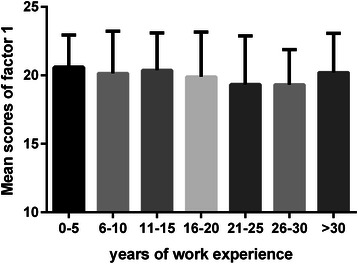
Mean scores of factor 1 relative to working years. Each bar indicates the mean scores of “learning beliefs and motivation” domain relative to the different years of rural physicians’ work experience. The Y error bar was displayed on top of each bar. Rural physicians with 21-30 working years had the lowest score in factor 1 demonstrated by multiple comparisons

**Fig. 3:**
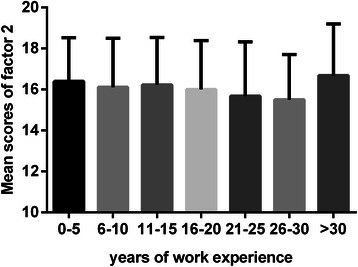
Mean scores of factor 2 relative to working years. Each bar indicates the mean scores of “attention to learning opportunities” domain relative to the different years of rural physicians’ work experience. The Y error bar was displayed on top of each bar. Rural physicians with 21-30 working years had the lowest score in factor 2 demonstrated by multiple comparisons

**Fig. 4:**
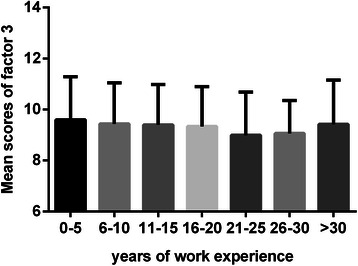
Mean scores of factor 3 relative to working years. Each bar indicates the mean scores of “technical skills in seeking information” domain relative to the different years of rural physicians’ work experience. The Y error bar was displayed on top of each bar. There was no significant difference in factor 3 in relation to the different years of work experience (*P* > 0.05)

We found significant differences in the learning beliefs and motivation domain (*F* = 3.131, *P* < 0.01) and the attention to learning opportunities domain (*F* = 2.995, *P* < 0.01) in relation to the different years of work experience (Table 5). Rural physicians who had 21-30 working years scored the lowest in the total mean score, factor 1 score and factor 2 score by multiple comparisons using the Student-Newman-Keuls test. The distribution of mean scores of each domain and the total score relative to the working years of the rural physicians are shown in Figs. 1, 2, 3 and 4.

We found that the orientation toward lifelong learning had a continuous decline in relation to an increase in working years and experience in medical practice until 30 years of work experience. Rural physicians with 0-30 working years showed significant impairment in factor 1, “learning beliefs and motivation”, and factor 2, “attention to learning opportunities”, as compared with rural physicians with more than 30 years of work experience, at which point there was a higher mean score of lifelong learning.

### Other correlates related to lifelong learning

Due to the small number of associate chief physicians and chief physicians, they were placed with attending physicians into one category, “practicing physicians”, when conducting analysis of correlates. Practicing physicians had significantly higher scores than assistant physicians in factor 1, “learning beliefs and motivation”, (*P* < 0.05) and factor 2, “attention to learning opportunities”, (*P* <0.05). Rural physicians with different career satisfaction levels showed significantly positive correlations in all domains, as well as the total score (*P* < 0.05). We found no significant association between total lifelong learning scores and the following variables: gender, age, and medical educational background (Table 6).

**Table 6:** Scores of rural physicians according to other impact factors of lifelong learning

Variables	Factor 1	Factor 2	Factor 3	Total
Professional titles				
Assistant physician	19.36 ± 3.05^a^	15.60 ± 2.41^a^	9.32 ± 1.59	44.28 ± 6.68^a^
Practicing physician	20.42 ± 2.02	16.54 ± 1.20	9.53 ± 2.00	46.49 ± 5.72
Career satisfaction				
Very unsatisfied	18.41 ± 4.25^b^	15.11 ± 3.14^b^	8.76 ± 1.98^b^	42.29 ± 8.67^b^
Relatively unsatisfied	17.75 ± 3.77	14.78 ± 2.60	8.73 ± 1.64	41.26 ± 6.70
Neutral	19.52 ± 3.14	15.61 ± 2.39	9.03 ± 1.57	44.17 ± 6.07
Relatively satisfied	20.31 ± 2.56	16.15 ± 2.14	9.32 ± 1.42	45.78 ± 5.35
Very satisfied	21.59 ± 2.37	17.62 ± 2.09	10.27 ± 1.56	49.48 ± 5.25

^a^ indicates significant difference of *t*-test, (*P* <0.05)

^b^ indicates significant difference of one-way ANOVA, (*P* <0.05)

## Discussion

Facing the present troubles of the capacity of medical practice not being able to meet the health care needs of rural residents, rural physicians need to cultivate awareness and habits of lifelong learning to maintain and to continuously improve the quality of medical treatment. To assess the current status of lifelong learning in rural physicians, JSPLL meets the key standards for validity and reliability of educational and psychological testing on Chinese rural physicians [36, 37]. Rural physicians generally performed better in the learning beliefs and motivation domain and worse in the technical skills in seeking information domain. Results showed that continuing medical education, years of work experience, career satisfaction, and professional title had a statistically significant link with lifelong learning (*P* <0.05).

The Cronbach’s α coefficient in this study (0.872) was similar to previous studies among physicians (Cronbach’s α = 0.86) [28] and medical students (Cronbach’s α = 0.77) [30]. Factor analysis in Chinese rural physicians showed a 3-factor solution, consisting of “learning beliefs and motivation,” “attention to learning opportunities,” and “technical skills in seeking information,” which showed similarities to the 3-factor patterns that emerged from the Hojat and Angela Wetzel studies [28, 30]. Additionally, the underlying construct of the JSPLL is consistent with the definition of the concept it is supposed to measure [21].

We found the mean score of lifelong learning for Chinese rural physicians (mean = 45.56, SD = 6.17) was lower than that was reported for American general clinicians (mean = 46.2, SD = 5.5) [28] and also much lower than Chinese urban physicians (mean = 46.97, SD = 5.5) [34]. There may be some explanations for this finding. First, in developing countries, higher-level urban hospitals do not only treat referred patients; tertiary hospitals^a^ are frequently the first point of contact with health services for many patients [38]. This may cause largescale urban hospitals to receive and treat more patients with serious illnesses than rural hospitals and community clinics, which present Chinese urban physicians with constant challenges and pushes them to continually learn and improve their skills. Additionally, physicians in urban areas usually undertake a heavier academic task driven by pressure of promotion and teaching for medical students. Empirical evidence shows a significant relationship between scores on lifelong learning and behavioral manifestations such as scientific activities and professional accomplishments [29]. Secondly, physicians in urban hospitals, as well as physicians in America usually have better library and network resources, and greater opportunities to participate in training, academic exchange, and scientific research [5], all of which are advantageous to lifelong learning of urban physicians and American general clinicians. According to the item mean score for each domain, rural physicians have lower orientation lifelong learning in the attention to learning opportunities domain and technical skill domain. This result suggests that providing rural physicians with more educational opportunities and abundant educational resources may be an effective strategy for improving their orientation to lifelong learning, instead of simply emphasizing the importance of lifelong learning for rural physicians.

We found that rural physicians who took part in continuing education more than 15 days a year had higher scores of lifelong learning in all three domains as compared to rural physicians who did not. Ryan J suggested that continuous professional development or continuing medical education is a fundamental component that lies along the continuum of lifelong learning [39]. Continuing medical education is an external manifestation of lifelong learning [22, 40]. The significant influence of different amounts of continuing education on lifelong learning of Chinese rural physicians in our study agrees with previous research by Simon [41] that people can improve their orientation toward lifelong learning by pursuing continuing education following completion of their undergraduate studies. Additionally, Frankford maintained that properly structured continuing medical education and equivalent functional activities are powerful engines to enhance physicians’ lifelong learning and commitment to medical professionalism [42]. Thus, continuing medical education should be encouraged to help rural physicians improve their orientation toward lifelong learning, and to develop their practical performance and medical competence.

In this study, rural physicians with 21-30 working years have the lowest total score of lifelong learning in comparison to other stages of working experience. From 0 to 30 working years, there is a decreasing trend in each five year phase. Similar findings have been reported in general studies conducted in the United Kingdom, Australia and Uganda [31, 43, 44], while Hojat et al reported the years after completing medical school had no significant correlative relationship within American physicians’ lifelong learning [28]. One possible explanation is that, in China, medical education places less emphasis on cultivating lifelong learning [45] than in the United States where Hojat et al conducted their studies. This results in a lack of awareness and understanding of the importance of lifelong learning during medical training. In addition, the Chinese system of continuing medical education is not all-encompassing for rural physicians in remote areas, and a specific re-certification policy was not strictly enforced to keep all rural physicians up-to-date on current national standards in their medical practice [5, 17, 46, 47]. Thus, the lack of a conscious personal approach and lack of institutional enforcement reflects and explains the significant impairment on “Learning beliefs and motivation” and “Attention to learning opportunities” of rural physicians. Focus should be placed on those rural physicians who are in their 3rd decade of medical practice to increase their internal attitude to lifelong learning.

However, a phenomenon that should be noted is the higher score of lifelong learning after 30 working years. Early career stress may have prevented physicians from developing their orientation toward lifelong learning. Physicians in their middle-age worked more hours and had the highest frequency of work-home conflicts, which led to work-life imbalance and both physical and emotional exhaustion [48]. Compared to the mid-career burning out, older physicians have less life and work stress and more job satisfaction at this point, which may mean more time and energy for learning [29, 49]. Furthermore, older, more experienced people have developed better verbal skills and judgment [50], which may add to their lifelong learning skills as they age. Crawford provided evidence and analyses on the possible psychological factors that support lifelong learning in older adults [51]. There is also a distinct feature in rural physicians who had worked more than 30 years. They usually began medical practice prior to the rural doctor training reforms in the early 1980s. This reform was established as a 3 year program^2^ to quickly train rural doctors with the most basic skills in order to meet the health care needs of the rural population. It lacks in professional development training [4, 6], which may be the reason for the decline in lifelong learning prior to 30 working years. For further research and understanding, a long-term survey with some specific open questions can help us deeper understand the trends of working years in relation to lifelong learning and find more appropriate solutions and strategies.

Practicing physicians had significantly higher total scores of lifelong learning than assistant physicians. As can be seen in the result, the difference was mainly seen in the lifelong learning beliefs, motivation, and attention to learning opportunities domains, which can be related to personal conscientiousness towards learning, while the technical skills aspect of lifelong learning did not differ significantly. The key difference between assistant physicians and attending (or practicing) physicians is that assistant physicians have a limited license that only grants them the right to prescribe in their rural jurisdictions and must work under the supervision of a practicing physician, whereas practicing physicians have more freedom to legally prescribe and more authority in the clinical setting [52]. In order to become a practicing physician, assistant physicians have to work at least 5 years in the clinical setting and take part in additional medical training, following which they must pass the National Medical Licensing Examination [52]. On the other hand, professional titles are also important indicators of professional accomplishments, including but not limited to published research or directing research programs. These activities and achievements cannot be successfully accomplished without an intrinsic motivation to learn and participation in vigorous learning activities [28, 29]. Therefore, when compared to current assistant physicians, practicing physicians may exhibit stronger beliefs, motivation, and attention towards lifelong learning. Previous studies also displayed relevant results in support of our findings [29, 53]. We assume the same for the Chinese rural physicians in our study.

Results show that the level of career satisfaction is significantly associated with the total score and three dimensions (motivation, attention, and skills) of lifelong learning. This result indicated that the physicians who have higher levels of satisfaction with their medical career are usually willing to conduct research studies and have self-awareness and motivation to continue their learning. Previous studies show career satisfaction is related to the extent of continuing professional development and demonstrate a significantly positive correlation between lifelong learning and career satisfaction [29, 54].

For this preliminary study, we conducted a cross-sectional study that may not have been an optimal way to assess variation tendency. Thus, the results of this study could act as indicators to guide future longitudinal studies on confirming the trend of influencing factors on rural physicians’ lifelong learning. It would be best to extend this study to a larger sample, especially since small sample sizes for some of the subgroups may have limited statistical strength. For example, in Table 6, there were only 18 physicians who were very unsatisfied with their career.

## Conclusions

In conclusion, our study suggests that the JSPLL performs well for assessing the orientation toward lifelong learning of Chinese rural physicians. Rural physicians had lower score than general physicians’ orientation to lifelong learning, and generally performed worse in the technical skills in seeking information domain and have a generally low level of lifelong learning. Providing rural physicians with more educational opportunities and abundant educational resources may be an effective strategy for improving their orientation to lifelong learning. Continuing medical education had a positive influence on lifelong learning, and orientation toward lifelong learning followed a trend of continuous decline between 0 and 30 years of work experience. Therefore, continuing medical education should be especially encouraged for Chinese rural physicians in their 3rd decade of medical practice. This will not only benefit the development of lifelong learning in Chinese rural physicians but will also improve their career satisfaction and increase professional accomplishments. The trends and influencing factors on lifelong learning in rural physicians act as strong indicators for further longitudinal studies and provide information for medical institutions and hospitals to strategize programs to improve lifelong learning for physicians.

## Additional file

Additional file 1: Figure S1. Diagram for the Chinese medical education system prior to 2014. The diagram illustrates four different main streams for cultivating a high school graduate to become a physician in Chinese medical education system. The blue full line arrow means the high school graduates who would like to choose physicians as their career must choose one of the four main streams to learn medicine depending on their scores of College Entrance Examination. The blue dotted line arrow means voluntary application process of medical students for further medical education. Notes for consideration: *Bachelor of Medicine is the Chinese equivalent to the British MBBS (Bachelor of Medicine and Bachelor of surgery); ^#^Doctor of Medicine is the Chinese equivalent to the American MD-PhD. (TIFF 1300 kb)

## Footnotes

1 According to the nation-wide hospital grading system formulated by the National Health and Family Planning Commission of China, Chinese hospitals are divided into three grades--primary, secondary, and tertiary referral hospitals. Each grade is a comprehensive indicator reflecting the scale of the hospital and its technical proficiency. Urban tertiary referral hospitals constitute the largest, most resource-intensive and most proficient facilities.

2 On the background of great shrink of rural physician during health-care reform in the early 1980s, the National Health and Family Planning Commission of China decided to address the shortage of health manpower in rural area by rapidly creating a large number of such college-level three-year programs. The curricula for these colleges were developed as simplification of the traditional five-year university programs, and focused on health problem specific to rural needs, in order to cultivate a large number of rural physicians as soon as possible. And now, these colleges gradually closed and replaced by five-year training program.
