# Supplementary figures and images for: Lifelong learning of Chinese rural physicians: preliminary psychometrics and influencing factors
Source: BMC Med Educ. 2015 Oct 30;15:192. doi: 10.1186/s12909-015-0460-9 (PMC4628275; doi:10.1186/s12909-015-0460-9)

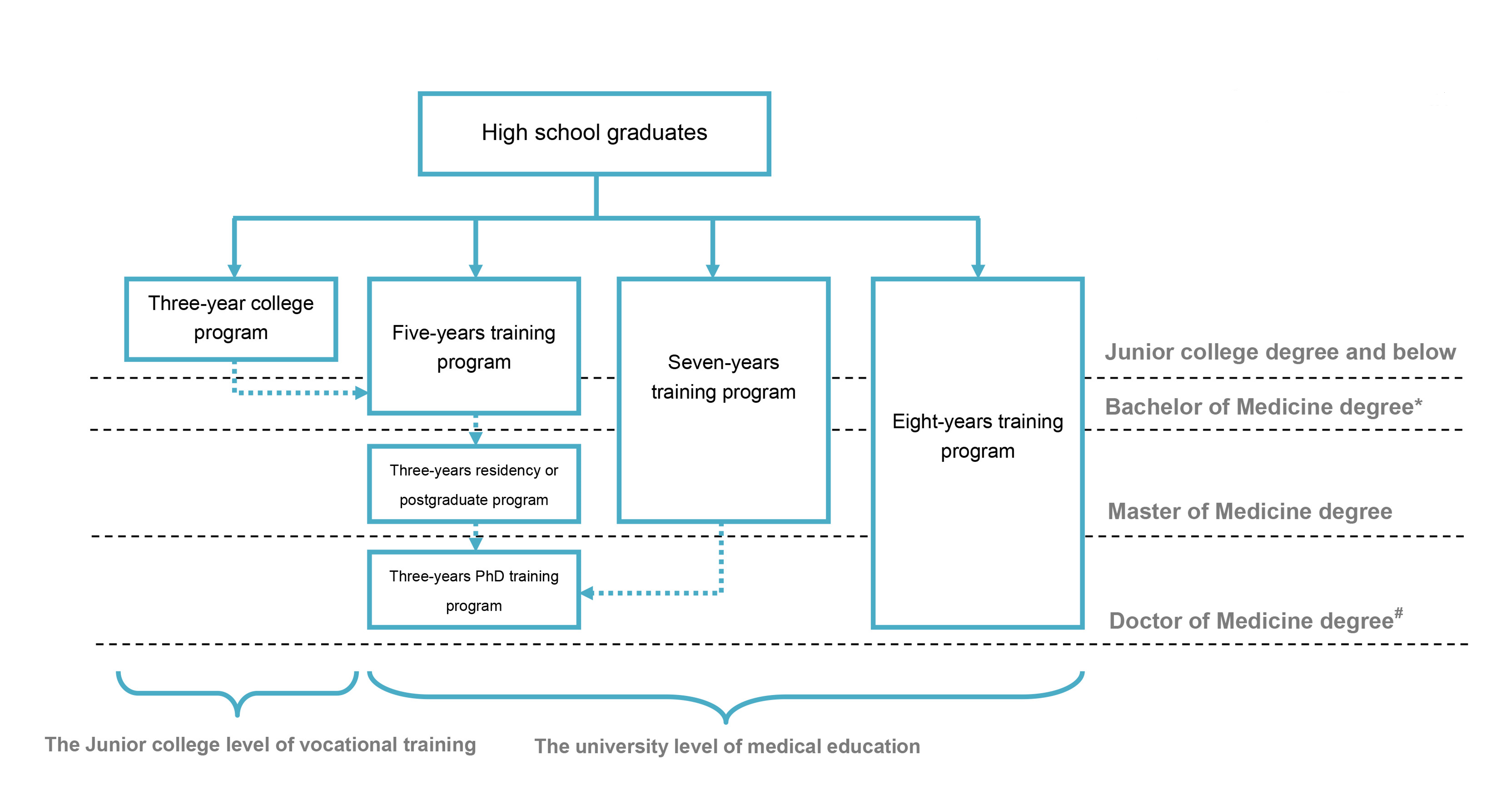

Supplement: Additional file 1: Figure S1. — Diagram for the Chinese medical education system prior to 2014. The diagram illustrates four different main streams for cultivating a high school graduate to become a physician in Chinese medical education system. The blue full line arrow means the high school graduates who would like to choose physicians as their career must choose one of the four main streams to learn medicine depending on their scores of College Entrance Examination. The blue dotted line arrow means voluntary application process of medical students for further medical education. Notes for consideration: *Bachelor of Medicine is the Chinese equivalent to the British MBBS (Bachelor of Medicine and Bachelor of surgery); #Doctor of Medicine is the Chinese equivalent to the American MD-PhD. (TIFF 1300 kb) [file 12909_2015_460_MOESM1_ESM.tiff]
